# Phage-Based Biosensing for Rapid and Specific Detection of *Staphylococcus aureus*

**DOI:** 10.3390/microorganisms11082098

**Published:** 2023-08-17

**Authors:** Ruining Li, Zhiwei Li, Chenxi Huang, Yifeng Ding, Jia Wang, Xiaohong Wang

**Affiliations:** 1 College of Food Science and Technology, Huazhong Agricultural University, Wuhan 430070, China; 2 Joint International Research Laboratory of Animal Health and Animal Food Safety, College of Veterinary Medicine, Southwest University, Chongqing 400715, China; 3 Key Laboratory of Environment Correlative Dietology, Huazhong Agricultural University, Wuhan 430070, China

**Keywords:** *Staphylococcus aureus*, *Staphylococcus* phage, magnetic beads, ATP bioluminescence assay

## Abstract

*Staphylococcus aureus* (*S. aureus*) is a major foodborne pathogen. Rapid and specific detection is crucial for controlling staphylococcal food poisoning. This study reported a *Staphylococcus* phage named LSA2302 showing great potential for applications in the rapid detection of *S. aureus*. Its biological characteristics were identified, including growth properties and stability under different pH and temperature conditions. The genomic analysis revealed that the phage has no genes associated with pathogenicity or drug resistance. Then, the phage-functionalized magnetic beads (pMB), serving as a biological recognition element, were integrated with ATP bioluminescence assays to establish a biosensing method for *S. aureus* detection. The pMB enrichment brought high specificity and a tenfold increase in analytical sensitivity during detection. The whole detection process could be completed within 30 min, with a broad linear range of 1 × 10^4^ to 1 × 10^8^ CFU/mL and a limit of detection (LOD) of 2.43 × 10^3^ CFU/mL. After a 2 h pre-cultivation, this method is capable of detecting bacteria as low as 1 CFU/mL. The recoveries of *S. aureus* in spiked skim milk and chicken samples were 81.07% to 99.17% and 86.98% to 104.62%, respectively. Our results indicated that phage-based biosensing can contribute to the detection of target pathogens in foods.

## 1. Introduction

*Staphylococcus aureus* (*S. aureus*) is a commensal bacterium that colonizes around 30% of people but also causes various infections, including bacteremia, endocarditis, and skin infections [1]. It is a major cause of food poisoning, contaminating diverse foods like milk, meat, salads, and bakery products due to its remarkable adaptability [2]. In the US, it leads to about 241,000 foodborne illness cases a year [3], and in China, it caused 4513 hospitalizations between 2003 and 2017 [4]. Antimicrobial resistance worsens the infection issue [5], highlighting the need for more effective *S. aureus* monitoring methods.

Various detection methods face limitations, hindering their broad application. For example, conventional culture-based methods are highly accurate but labor-intensive and time-consuming [6,7]. The PCR-based method offers good analytical specificity and sensitivity but requires specialized equipment and skilled personnel. It also causes potential false positives from unexpected pollutants and dead bacteria [8]. The immunological method has exceptional antigen-antibody binding ability but suffers from long operating times, cumbersome steps, and difficulty in preparing monoclonal antibodies [9]. Despite their popularity, implementing these methods for onsite pathogen monitoring remains challenging. Thus, alternative tools are needed for rapid, specific, and sensitive pathogen detection.

Biosensors are appealing alternatives to conventional pathogen detection methods, offering short assay times, high specificity, easy sample preparation, miniaturization, and onsite monitoring. Bacteriophages are popular recognition elements due to their advantages of stability, easy immobilization, and strict specificity to targets [10]. They maintain specificity even in mixed bacterial systems and are abundant in the environment [11]. Adenosine triphosphate (ATP) bioluminescence is highly efficient for onsite detection of live cells. It responds to live bacteria by measuring ATP levels, which remain relatively constant inside cells and dissipate quickly in the environment [12]. However, different ATP sources can interfere, requiring a pretreatment step.

Here, we reported a *Staphylococcus* phage LSA2302 isolated from sewage using *S. aureus* ATCC25923 as the host. The phage’s biological and genomic characteristics were studied. Next, phage LSA2302 was combined with magnetic beads to create a magnetic recognition element for specific enrichment of target *S. aureus*. This phage-based bio-probe was integrated with the ATP bioluminescence assay to establish a biosensor for rapid and specific *S. aureus* detection. The biosensor’s performance was evaluated for analytical specificity and sensitivity under various conditions. Its diagnostic applicability was tested in skim milk and chicken samples, revealing the potential of phage-functionalized magnetic beads for fast and specific detection of foodborne pathogens.

## 2. Materials and Methods

### 2.1. Chemical and Materials

The Lysogeny Broth (LB) and Baird-Parker Agar Base were procured from Hopebio (Qingdao, China), while 1-ethyl-3-(3-dimethylaminopropyl) carbodiimide hydrochloride (EDC), N-hydroxysulfosuccinimide sodium salt (NHS), and phosphate-buffered saline were acquired from Macleans Biochemical Technology Co., Ltd. (Shanghai, China). Additionally, 4-morpholineethanesulfonic acid (≥99%), D-luciferin, and recombinant luciferase were purchased from Aladdin Industrial Corporation (Shanghai, China), whereas bovine serum albumin (BSA) and cetyltrimethylammonium bromide (CTAB) were obtained from Solarbio Science & Technology Co., Ltd. (Beijing, China). The average size of 1 μm carboxylated magnetic beads was procured from PuriMag Biotechnology Ltd. (Xiamen, China), and black 96-well polystyrene microplates were obtained from Costar (Costar Inc., Milpitas, CA, USA). Skimmed milk powder was acquired from BD Company (Franklin Lakes, NJ, USA).

### 2.2. Bacterial Strains and Culture Condition

Bacterial strains used in the experiments are listed in Table S1. Without specification, bacterial strains were stored in 20% (*v*/*v*) glycerol at −80 °C and cultured in LB at 37 °C when needed. *Vibrio parahaemolyticus* was cultured in LB supplemented with 3% sodium chloride. 

### 2.3. Isolation and Purification of Phage LSA2302

Phage LSA2302 was isolated from sewage collected at a market located in Wuhan, China, using *S. aureus* ATCC 25923 as the host strain. To this end, the sewage sample was centrifuged at 10,000× *g* for 15 min. Subsequently, the supernatant was filtered with a 0.22 μm membrane. The filtrate and *S. aureus* suspension were then added to LB and incubated at 37 °C for 18–20 h. The presence of phage was verified using the double-layer agar method [13]. The individual plaques were picked for purification until the lytic plaque became homogeneous. The purified phage was preserved at −80 °C with 20% glycerol.

### 2.4. Host Range Determination

The host range of LSA2302 was determined by the spot method [14]. Briefly, 100 μL of a bacterial suspension was mixed with LB containing 0.7% agar, and the mixture was then poured onto the solid medium plate (1.4% agar). Next, 10 μL of phage suspension was dropped onto the mixed plate, and the plate was then inverted and incubated at 37 °C for 6–8 h. The host range of LSA2302 was determined by observing the transparency of the phage spots.

### 2.5. Morphological and Structural Protein Analysis 

The morphological characterization of the phage was observed via transmission electron microscopy (TEM). The phage LSA2302 suspension (10^9^ PFU/mL) was ultracentrifuged at 40,000× *g* for 1 h and then resuspended in 0.1 mol/L ammonium acetate. Subsequently, the copper mesh was submerged in the phage suspension for 10 min and stained with a 2% (*w*/*v*) solution of phosphotungstic acid (pH 7.0) for 10 min. The morphological characterization of phage LSA2302 was observed using a transmission electron microscope (Hitachi H-7000FA, Tokyo, Japan) and calculated with Digital Micrograph Demo 3.9.1. For structural protein analysis, the phage particles were extracted and enriched using a previously established method [15]. Then the phage protein was analyzed via sodium dodecyl sulfate polyacrylamide gel electrophoresis (SDS-PAGE).

### 2.6. The Optimal Multiplicity of Infection 

The multiplicity of infection (MOI) represents the ratio of phages to host bacteria [16]. Phage dilutions were blended with an equal volume of *S. aureus* ATCC25923 suspensions (10^6^ CFU/mL) at varying MOIs (100, 10, 1, 0.1, 0.01). The mixtures were then incubated at 37 °C for 3.5 h, followed by centrifugation at 10,000× *g* for 2 min. The phage titer in supernatants was calculated, and the optimal MOI corresponded to the highest titer.

### 2.7. Adsorption Rate 

An equal volume of the host strain suspension was added to the phage dilution (5 mL) according to the optimal MOI (=10), and the resulting mixture was incubated at 37 °C. At 0 min and every 3 min for a total of 30 min, 300 μL of the culture solution was collected and centrifuged at 10,000× *g* for 2 min. The titer of unadsorbed phage in the supernatant was examined with the double-layer agar method. The adsorption rate was calculated using the following formula: adsorption rate (%) = (initial phage titer − free phage titer)/initial phage titer.

### 2.8. One-Step Growth Curve

An equal volume of *S. aureus* suspension at optimal MOI (=10) was added to the phage dilution, which was then incubated at 37 °C for 15 min and subsequently centrifuged at 8000× *g* for 2 min. The resultant precipitate was resuspended in 10 mL of fresh LB and further cultured. A 300 μL aliquot of the culture solution was collected every 10 min for 180 min and subsequently centrifuged at 10,000× *g* for 2 min. The titer of the supernatant was determined using the double-layer agar method. The relative burst size was then calculated using the formula: relative burst size = final phage titer/initial host concentration.

### 2.9. Thermal and pH Stability 

The thermal and pH stability of phage LSA2302 was assessed as previously reported, with slight modifications [13]. For thermal stability, 1 mL of phage dilutions (10^7^ PFU/mL) were subjected to treatment at temperatures ranging from 30 to 80 °C for 30 and 60 min. For pH stability, 100 μL of the phage dilution (10^7^ PFU/mL) was mixed in 900 μL of phosphate buffer saline (PBS) at pH levels ranging from 2.0 to 12.0 for a duration of 2 h. Following treatment, the titer of each sample was determined using the double-layer agar method.

### 2.10. Lytic Curve 

Phage dilutions (100 μL) were blended with an equal volume of *S. aureus* ATCC25923 suspensions at varying MOIs (1000, 100, 10, 1, 0.1, 0.01, 0.001). The mixtures were then incubated in a 96-well plate at 37 °C for 10 h. Absorbance at 600 nm was measured at a 1-h interval. The bacteria suspension (200 μL) and LB medium (200 μL) served as positive and negative controls, respectively. 

### 2.11. Genomic Analysis

The DNA of LSA2302 was extracted according to a previously validated method [17], followed by the measurement of concentration using a Qubit fluorometer (Thermo Fisher, Waltham, MA, USA). The phage genome was sequenced on the Illumina HiSeq platform (Illumina, San Diego, CA, USA) and assembled with MicrobeTrakr Plus 0.9.1 [18]. Putative open reading frames (ORFs) were predicted with the online prediction tools RAST (https://rast.nmpdr.org/, accessed in 1 November 2022, applies to other websites mentioned below) [19], GeneMarkS (http://topaz.gatech.edu/GeneMark/genemarks.cgi) [20], and ORF Finder (https://www.ncbi.nlm.nih.gov/orffinder/). The function of putative ORFs was manually annotated using the NCBI online tool BLASTp and the Conserved Domain Database (CDD, https://www.ncbi.nlm.nih.gov/cdd). Genes encoding tRNAs were identified by tRNAscan-SE [21]. The gene map for LSA2302 was generated by Proksee (https://proksee.ca/). The phylogenetic tree was constructed using MEGA7 based on the terminase large subunit. Antibiotic resistance genes and virulence genes were identified using the Comprehensive Antibiotic Resistance Database (CARD, https://card.c.ca/analyze/rgi) [22] and the virulence factor database (VFDB, http://www.mgc.ac.cn/VFs/) [23], respectively.

### 2.12. Functionalization of Magnetic Beads by Phage LSA2302

Phage LSA2302-functionalized magnetic beads (pMB) were assembled according to the previously reported method with some modifications [24]. In brief, 500 μg of sufficiently dispersed (by ultrasound) magnetic beads (10 μg/μL, 1 μm in diameter) were washed twice with 200 μL of MES buffer (50 mM, pH 5.0) and then resuspended in 200 μL of PBS supplemented with EDC (50 mg/mL) and NHS (50 mg/mL). The resuspension was incubated on a rotary mixer (TR-02U, Crystal, TX, USA) for 30 min to ensure adequate reactions. Subsequently, excess EDC and NHS were removed via washing with PBS. The magnetic beads were then conjugated with the phage suspension (10^9^ PFU/mL) for a duration of 2 h. The remaining binding sites on the surface of the magnetic beads were blocked with 5% (*w*/*v*) bovine serum albumin (BSA). The functionalized magnetic beads were characterized using the spot method [14] and TEM to evaluate their host-binding ability and were stored in 2% BSA at 4 °C until use.

### 2.13. Detection of S. aureus by pMB Combined with ATP Bioluminescence

The standard detection procedure was established as follows: The overnight culture of *S. aureus* ATCC25923 was diluted to a final concentration of 10^5^ CFU/mL. Then the bacterial dilution (1 mL) was mixed with 60 μg of pMB (2 μg/μL) and incubated at 25 °C for 15 min. The resultant pMB-bearing target *S. aureus* was magnetically separated for 2–3 min and washed with PBS. Subsequently, separated pMB was resuspended in 100 μL of 0.85% NaCl and treated with 0.02% (*w*/*v*) CTAB for 1 min to disrupt the cells of *S. aureus*. The resulting samples were transferred to a black opaque 96-well plate and mixed with an equal volume (50 μL) of pre-equilibrated luciferin-luciferase mixed liquid (the concentrations of luciferin and luciferase were 0.20 mg/mL and 0.16 mg/mL, respectively). Finally, luminescence was immediately measured using the EnSpire Multilabel Reader 2300 (PerkinElmer Inc., Waltham, MA, USA).

### 2.14. Detection Performance of pMB under Different Conditions

The effect of pMB amount (20, 40, 60, 80, 100, and 150 μg), capture time (5, 10, 15, 20, 25, and 30 min), capture temperature (4, 25, and 37 °C), bacterial concentration (10^3^–10^7^ CFU/mL), lysis time (1–5 min), and CTAB concentration (0.005%, 0.01%, 0.02%, 0.05%, and 0.1%) on the detection performance of the pMB biosensor was studied. Without specification, except for the tested parameter, all conditions were maintained as described in the established standard detection procedure. For the effects of pMB amount and capture time, 37 °C was used as the capture temperature. The capture rate was calculated by using the following equation: capture rate (%) = (1 − N1/N2) × 100%, where N1 represents the count of uncaptured bacteria and N2 represents the initial bacterial count.

### 2.15. Analytical Sensitivity and Specificity of pMB-Based Detection of S. aureus

Without specification, all conditions were maintained as described in the established standard detection procedure. For analytical sensitivity, the gradient concentration of the *S. aureus* ATCC25923 dilution was integrated into the standard detection procedure to generate the calibration curve and calculate the limit of detection (LOD). For analytical specificity, both target *S. aureus* bacteria and non-target bacteria, including those from *Escherichia coli*, *Listeria monocytogenes*, *Vibrio parahaemolyticus*, *Salmonella*, and *Pseudomonas aeruginosa*, were integrated into the standard detection procedure. For different bacteria, respective capture rates and ATP bioluminescence responses were determined. 

### 2.16. Detection of S. aureus in Spiked Samples

The chicken was processed according to the previous method [25]. Skim milk powder (10 g) was dissolved in 100 mL of distilled water and sterilized at 65 °C for 30 min. The chicken and milk samples were added with S. *aureus* dilutions at final concentrations of 10^5^, 10^6^, and 10^7^ CFU/mL, respectively. For pre-cultivated samples, 1 CFU/mL of the bacterial dilution was cultured for 2 h before detection. The pMB-based detection was conducted according to the established standard detection procedure. The conventional culture-based method was used as a reference to calculate recovery rates.

### 2.17. Statistical Analysis

The experiments were independently performed in triplicate. The data were assessed for statistical significance using a *t*-test and expressed as mean ± standard deviation. The level of statistical significance was designated as *p* < 0.05.

## 3. Results

### 3.1. Biological Characteristics of Phage LSA2302

**Figure 1:**
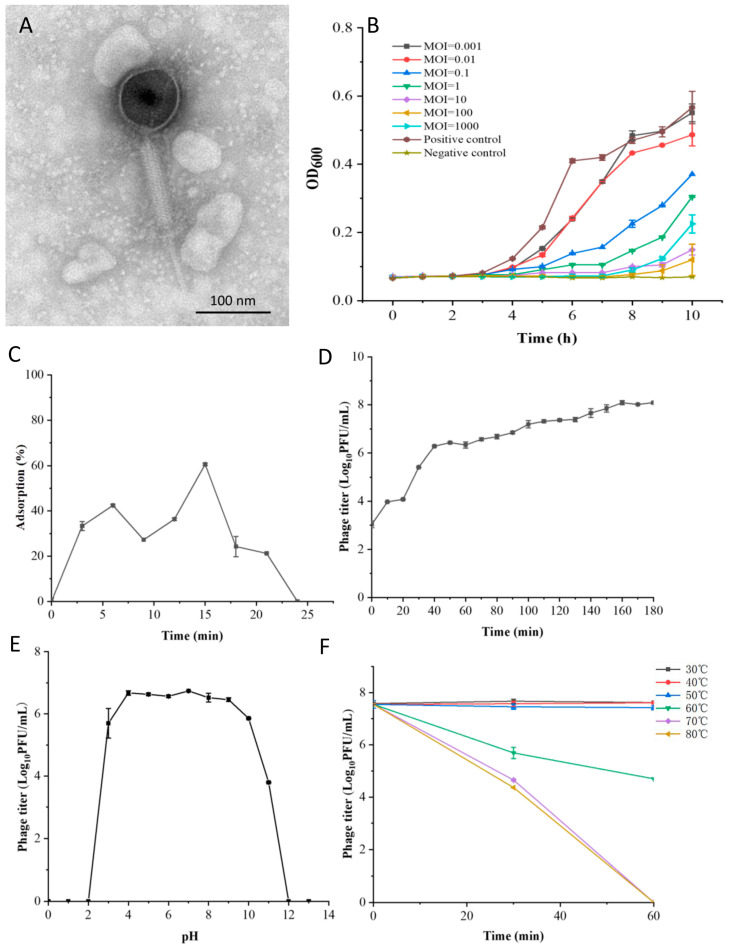
Biological characteristics of phage LSA2302. (**A**) TEM image of phage LSA2302 with a bar representing a magnification size of 100 nm. (**B**) LSA2302 inhibition of *S. aureus* ATCC 25923 growth at MOIs ranging from 0.001 to 1000. (**C**) Adsorption rate. (**D**) One-step growth curve. (**E**) Phage viability under different pH conditions. (**F**) Phage viability at different temperatures.

TEM analysis showed that phage LSA2302 had an isometric, icosahedral head of approximately 100.6 nm in diameter and a long, contractile tail with a baseplate and small collar (Figure 1A), indicating it is likely to belong to the *Herelleviridae* family [26]. SDS-PAGE analysis revealed ten bands ranging from 10 to 180 kDa, with the most abundant band at approximately 55 kDa, likely corresponding to the major capsid protein (Figure S1A). LSA2302 showed high specificity for *S. aureus*, with no lytic activity against other tested bacteria, but it lysed 18 out of 27 (66.67%) tested *S. aureus* strains (Figure S1B). An adequate inhibition of *S. aureus* growth was observed at MOIs of 10, 100, and 1000, completely abolishing bacterial growth until 7 h after incubation. However, at an MOI of 1000, bacterial growth rebounded more noticeably from 8 to 10 h compared to MOIs of 10 and 100. The inhibitory effect was weak at MOIs of 0.001 and 0.01 (Figure 1B).

The growth characteristics of LSA2302 were studied using its host strain, *S. aureus* ATCC25923, at an optimal MOI of 10 (Table S2). The maximum adsorption rate of 60.61% was detected at 15 min after incubation, followed by a sharp decrease from 15 to 24 min (Figure 1C). The one-step growth curve indicated a latent period of 20 min and exponential growth from 20 to 160 min with a burst size of 38.46 PFU/cell (Figure 1D). LSA2302 remained active at pH 4 to 9, but was partially impaired at pH 3, 10, and 11, and completely inactive at pH 2 and 12 (Figure 1E). At temperatures lower than 50 °C, LSA2302 remained active with consistently high titers, but viability was partly attenuated after incubation at temperatures higher than 70 °C for 30 min and completely lost for 60 min (Figure 1F). 

### 3.2. Genomic Features of Phage LSA2302 

**Figure 2:**
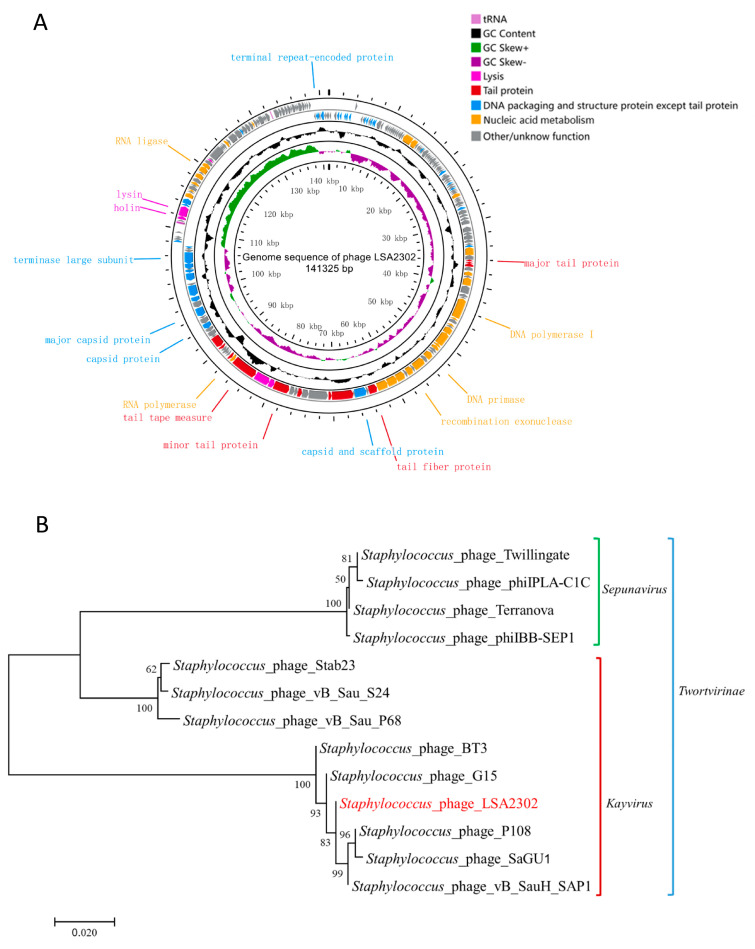
Genomic analysis of phage LSA2302. (**A**) Circular genome map of LSA2302, with the function of representative ORFs labeled, where the coding regions are indicated by the arrows illustrating the direction of transcription. From outside to inside: ORFs, GC content, GC skew, the scale. (**B**) Phylogeny of phages based on the sequences of the terminase large subunit. Phage LSA2302 is highlighted in red.

The key information of LSA2302’s genome (e.g., linear dsDNA, 141,325 bp, 30.20% GC content) was summarized in Table S3. It had 222 predicted ORFs, with likely 89 functional and 133 putative proteins. Functional ORFs were categorized into four modules: lysis, tail protein, DNA packaging and structure protein, and nucleic acid metabolism (Figure 2A; Table S4). The phage genome contained three tRNAs (tRNA-Met, tRNA-Asp, and tRNA-Phe) but no virulence or antibiotic-resistance genes, ensuring its safety for use in foods. Phylogenetic analysis based on the terminase large subunit showed LSA2302 closely clustered with phages from the *Kayvirus* genus of the *Herelleviridae* family (Figure 2B).

### 3.3. Assembly and Characterization of Phage-Functionalized Magnetic Beads

**Figure 3:**
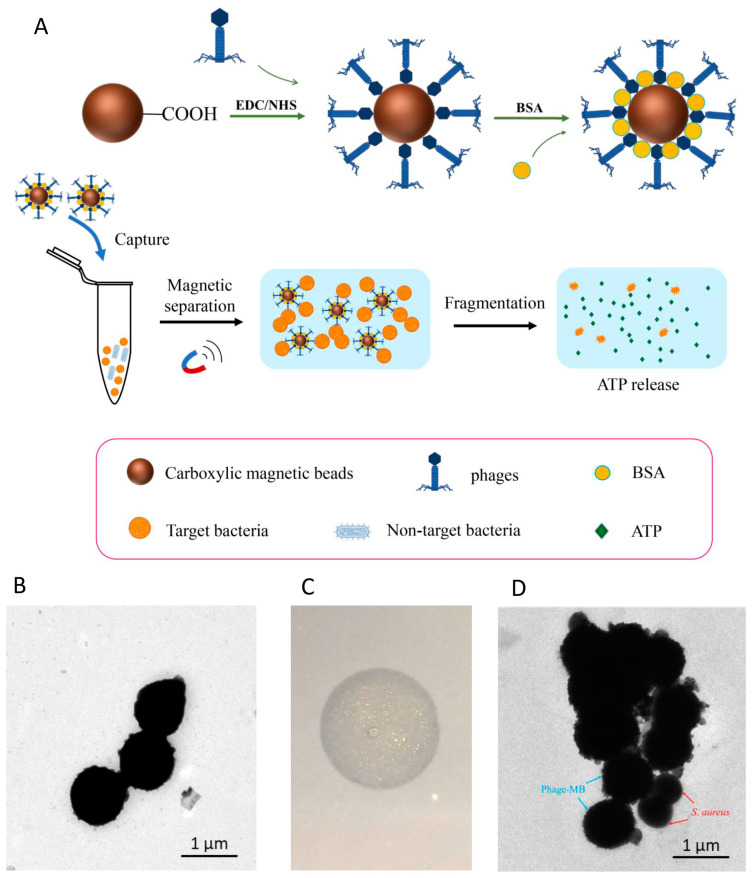
Functionalization of magnetic beads by phage LSA2302. (**A**) Schematic illustration of the assembly of phage-functionalized magnetic beads (pMB) and pMB-based capture of target bacteria. (**B**) TEM image of magnetic beads with a bar representing a magnification size of 1 μm. (**C**) The lytic zone induced by pMB on the lawn of *S. aureus* ATCC 25923. (**D**) TEM image of pMB bearing *S. aureus* ATCC 25923 with a bar representing a magnification size of 1 μm.

Figure 3A illustrates the assembly of phage LSA2302-functionalized magnetic beads (pMB) and pMB-based detection of target *S. aureus*. Carboxylated magnetic beads (Figure 3B) were activated through EDC/NHS activation and then conjugated with phages via stable covalent bonds. The phage-bead conjugates were incubated with BSA to prevent non-specific adsorption. After capturing the target bacteria, pMB with the target was collected, and the cells were lysed using CTAB, releasing intracellular ATP, which was quantified using the luciferase/luciferin reaction. The functional activity of phages bound to the beads was confirmed by the clear lytic zone on the *S. aureus* plate (Figure 3C). TEM observation also demonstrated successful capture of the target *S. aureus* strain by pMB (Figure 3D).

### 3.4. Optimization of Conditions for pMB-Based Detection of S. aureus

**Figure 4:**
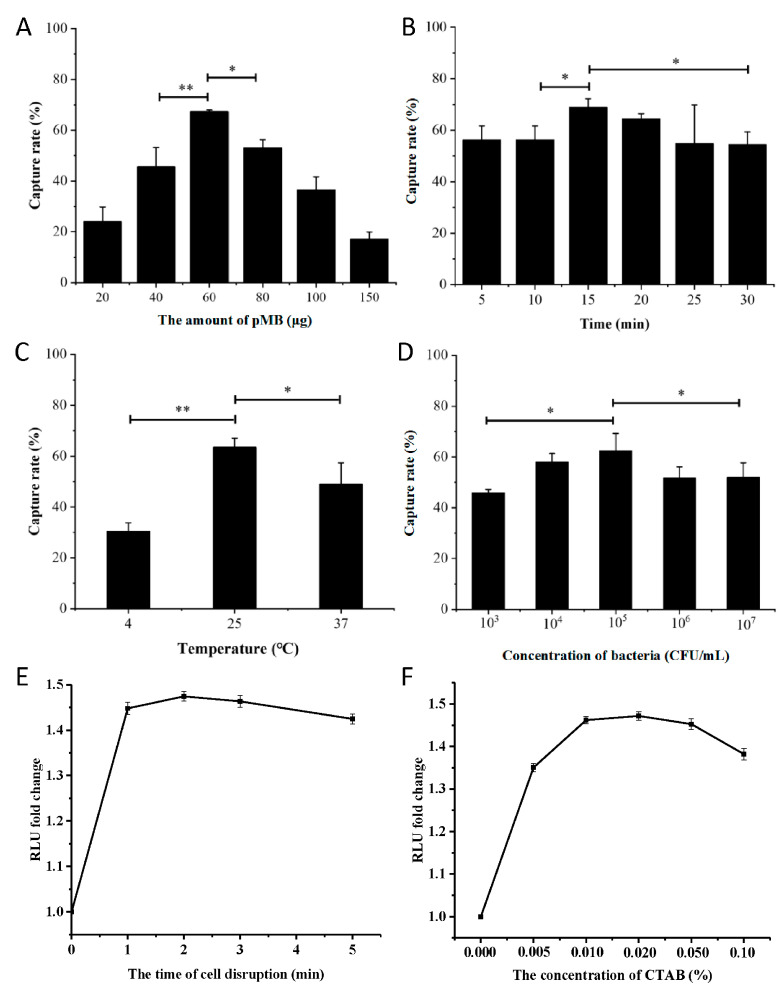
Optimization of the condition for pMB-based detection of *S. aureus*. (**A**) The effect of the pMB amount. (**B**) The effect of capture time. (**C**) The effect of capture temperature. (**D**) The effect of bacterial concentration. (**E**) The effect of lysis time. (**F**) The effect of CTAB concentration. * *p* < 0.05, ** *p* < 0.01.

The capture rate of the target by pMB was influenced by several parameters. The most effective capture (67.3%) occurred at a pMB amount of 60 μg within the range of 20 to 150 μg. Increasing the pMB amount beyond 60 μg resulted in a gradual decrease in capture rate (Figure 4A). Similar patterns were observed for the capture rate in response to capture time, capture temperature, and bacterial concentration (Figure 4B–D). The best pMB-based capture was achieved at a capture time of 15 min, a capture temperature of 25 °C, and a bacterial concentration of 10^5^ CFU/mL (Figure 4B–D). Additionally, cell lysis for 1 min generated an adequate signal for quantification, and no significant increase in signal intensity was observed with prolonged lysis time (Figure 4E). The optimal ATP bioluminescence response was observed at a CTAB concentration of 0.02% (Figure 4F).

### 3.5. Analytical Sensitivity and Specificity of pMB-Based Detection of S. aureus

**Figure 5:**
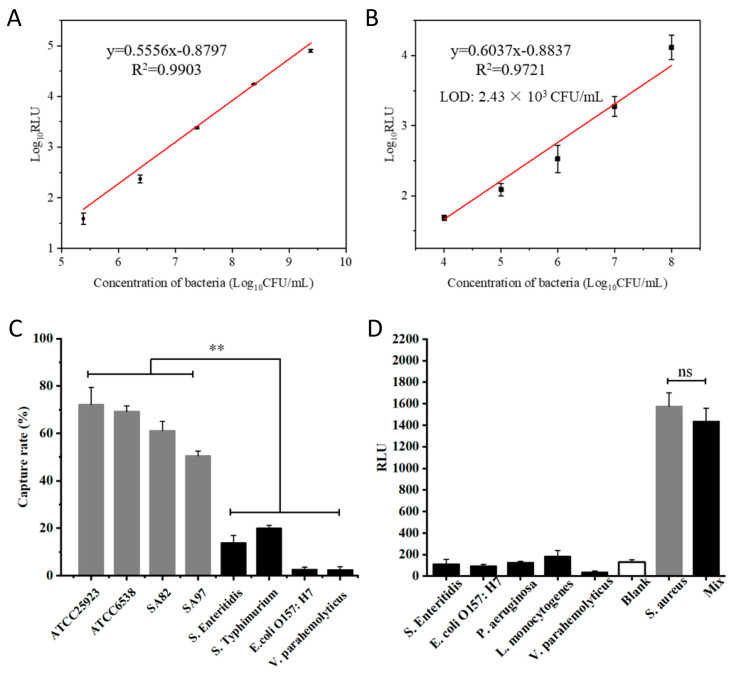
Analytical sensitivity and specificity of pMB-based detection of *S. aureus*. (**A**) The calibration curve of the ATP bioluminescence response to different concentrations of *S. aureus* without pMB enrichment. (**B**) The calibration curve of the ATP bioluminescence response to different concentrations of *S. aureus* after pMB enrichment. (**C**) Capture rates of different types of bacteria captured by pMB. (**D**) ATP bioluminescence responses to different types of bacteria after pMB enrichment. Mix: target *S. aureus* mixed with an equal concentration of *Salmonella*. ** *p* < 0.01, ns: not significant.

The pMB-based detection approach’s analytical sensitivity was assessed by measuring ATP bioluminescence responses to various concentrations of target bacteria. Without pMB enrichment, the ATP bioluminescence assay showed a linear range of 10^5^ to 10^9^ CFU/mL, but it was insensitive below 10^5^ CFU/mL (Figure 5A). With pMB enrichment, the analytical sensitivity of the method improved up to 10-fold, with a linear range of 10^4^ to 10^8^ CFU/mL and a limit of detection (LOD) of 2.43 × 10^3^ CFU/mL (Figure 5B). Additionally, pMB enrichment provided high analytical specificity to the approach. The capture rate for target *S. aureus* strains ranged from 50.49% to 72.16%, while interfering bacteria had capture rates lower than 20% (Figure 5C). ATP bioluminescence responses also showed similar analytical specificity, with the target *S. aureus* strain generating a significantly stronger signal than interfering bacteria. Even when the target *S. aureus* strain was mixed with an equal concentration of *Salmonella*, the signal was not significantly impaired (Figure 5D).

### 3.6. Evaluation of pMB-Based Detection of S. aureus in Spiked Samples

Recovery tests were conducted in skim milk and chicken samples spiked with target *S. aureus* at three different concentrations to assess the reliability and robustness of the pMB-based detection approach. In skim milk, the recovery and relative standard deviation (RSD) values ranged from 81.07% to 99.17% and 1.13% to 2.06%, respectively. In chicken samples, the values were 86.98% to 104.62% and 1.64% to 3.48%, respectively (Table 1). After a 2 h pre-cultivation, the method could detect bacteria as low as 1 CFU/mL, with recovery and RSD values of 98.57% and 4.63%, respectively (Table 1). These acceptable values demonstrate the applicability of the pMB-based detection approach in complicated sample matrices. Compared to other reported methods, pMB-based biosensing offers a significant advantage in terms of detection time (Table S5).

## 4. Discussion

The initial stage of phage infection is attachment to the host cell surface, known as adsorption [27]. Phage LSA2302 achieved peak adsorption at 15 min post-inoculation, which corresponds to the optimal capture time for pMB-based detection. This implies that the conjugation of phages with magnetic beads did not hinder phage-host interaction; at least the influence is undetectable in this context. This is confirmed by LSA2302 pMB’s active ability to lyse target bacteria. 

LSA2302 has several characteristics, such as specificity and heat and pH tolerance, making it a promising agent for easy *S. aureus* detection and diagnosis. Firstly, its lytic activity is specific to *S. aureus* strains without cross-reactions with other types, ensuring high specificity in pMB-based detection and avoiding interference by non-target strains. Additionally, LSA2302 remains stable at various temperatures, as 50 °C for 60 min did not reduce its viable phage titers significantly, unlike other *S. aureus* phages [28,29]. It also exhibits comparable tolerance to acidic and alkaline conditions, enabling its use in complex matrices with varying pH levels, making it particularly suitable for food industry applications since pH levels in food samples can vary widely [30].

ATP serves as the major energy carrier in living cells, and its constant content makes it a quantitative biomarker for microbial enumeration. The ATP bioluminescence assay utilizes luciferase to convert ATP to AMP, emitting light that can be measured by RLU levels, providing rapid results within minutes [31]. We integrated the ATP bioluminescence assay and LSA2302 pMB enrichment for rapid and specific *S. aureus* quantification within 30 min. It exhibits a significant advantage in terms of detection time. Rapid and convenient detection of foodborne pathogens has always been highly anticipated. When evaluating and selecting tests for detecting foodborne pathogens, the primary criterion to follow is the speed of detection [32]. Furthermore, after pre-culturing for 2 h, our method can detect target strains as low as 1 CFU/mL, demonstrating that phage-based biosensing is an effective tool for pathogen detection. 

The pMB-based method established in this study exhibits limitations in terms of diagnostic sensitivity, which arise from the inherent characteristics of phage LSA2302. It lysed 18 out of 27 (66.67%) tested *S. aureus* strains while showing no lytic activity against other types of bacteria. This host spectrum certainly helps prevent false positives due to cross-reactivity, but it may simultaneously restrict the application of LSA2302 as it cannot recognize all tested *S. aureus* strains. It is important to note that such limitations stemming from the characteristics of LSA2302 do not confine applying the phage-based method to detect foodborne pathogens. This study primarily established and evaluated a method that combines phages with ATP bioluminescence assays for the rapid detection of foodborne pathogens. To further improve the pMB-based method, a crucial aspect involves the continued search for and testing of phage candidates with better host spectra, which is a fundamental element of all phage-based diagnostic and therapeutic strategies.

Our results revealed the impact of magnetic bead dosage, incubation time and temperature, and bacterial concentration on LSA2302 pMB-based detection efficiency. Increasing pMB amounts enhanced capture efficiency due to more binding sites, but excessive pMB accumulation might weaken its interaction with bacteria, leading to a decline in capture rate. The specificity of pMB-based biosensing resembles antibody-based immunological methods, but phage production is faster and more cost-effective. Phage-based biosensing typically takes less than 1 h since prolonged incubation may cause cell lysis, which could explain why capture efficiency declined with longer incubation times [33]. An optimal temperature of 25 °C was found for LSA2302 pMB-based biosensing, which is lower than previously reported optimal temperatures of phage or ATP bioluminescence-based methods, enabling its use at room temperature and facilitating its application [34,35].

Matrix spiking evaluates the analytical approach, and acceptable results boost our confidence in the accuracy and validity of LSA2302 pMB-based biosensing in complex matrices. Milk and chicken samples were selected for recovery tests due to their reported high prevalence of multidrug-resistant *S. aureus* contamination [36,37]. The pMB-based detection approach demonstrated comparable recovery and RSD values in complicated food matrices compared to other phage-based methods but with significantly shorter detection times [38,39].

## 5. Conclusions

In this study, *Staphylococcus* phage LSA2302 was characterized biologically and genomically. It was combined with magnetic beads to create functional pMB as recognition elements, integrated with ATP bioluminescence assays for rapid *S. aureus* detection. LSA2302 pMB provided high analytical specificity and increased analytical sensitivity. Validation in spiked samples showed acceptable recovery and RSD values, indicating its potential for rapid and specific *S. aureus* detection in foods.

## Figures and Tables

**Table 1:** Recovery test in spiked samples and after pre-cultivation.

Sample	Standard Method	pMB	Recovery (%)	RSD ^a^ (%)
Skim milk	1.20 × 10^7^	1.19 × 10^7^	99.17	1.93
	5.60 × 10^6^	4.54 × 10^6^	81.07	1.13
	4.10 × 10^5^	3.68 × 10^5^	89.76	2.06
Chicken	1.70 × 10^7^	1.60 × 10^7^	94.12	2.85
	5.30 × 10^6^	4.61 × 10^6^	86.98	1.64
	1.30 × 10^5^	1.36 × 10^5^	104.62	3.48
pre-cultivation ^b^	2.10 × 10^5^	2.07 × 10^5 c^	98.57	4.63

^a^: RSD—relative standard deviation; ^b^: 1 CFU/mL of the bacterial dilution was cultured for 2 h, followed by the quantification via both conventional culture-based and pMB-based methods; ^c^: Log_10_RLU = 2.33; Calculated using y = 0.6037x − 0.8837.

## Data Availability

The data presented in this study are available on request from the corresponding author.

## Supplementary Materials

The following supporting information can be downloaded at: https://www.mdpi.com/article/10.3390/microorganisms11082098/s1, Figure S1: (A) Structural protein analysis by SDS-PAGE. M: marker; lane 1: phage LSA2302. (B) The host range of phage LSA2302 with scoring on lytic zones: “2” denotes strong intensity (clear plaque); “1” denotes weak intensity (opaque plaque); “0” denotes no lytic activity (no plaque); Table S1: Bacterial strains used in this study and the host range of phage LSA2302; Table S2: The optimal multiplicity of infection (MOI) of phage LSA2302; Table S3: Genomic features of phage LSA2302; Table S4: Predicted ORFs in the phage LSA2302 genome; Table S5: The comparison of pMB-based biosensing with other previously reported methods for *Staphylococcus aureus* detection.
