# Supplementary material for: Phage-Based Biosensing for Rapid and Specific Detection of Staphylococcus aureus
Source: Microorganisms. 2023 Aug 17;11(8):2098. doi: 10.3390/microorganisms11082098 (PMC10458643; doi:10.3390/microorganisms11082098)
Supplement: Supplementary file 1 [file microorganisms-11-02098-s001.zip › Figure S1.pdf]

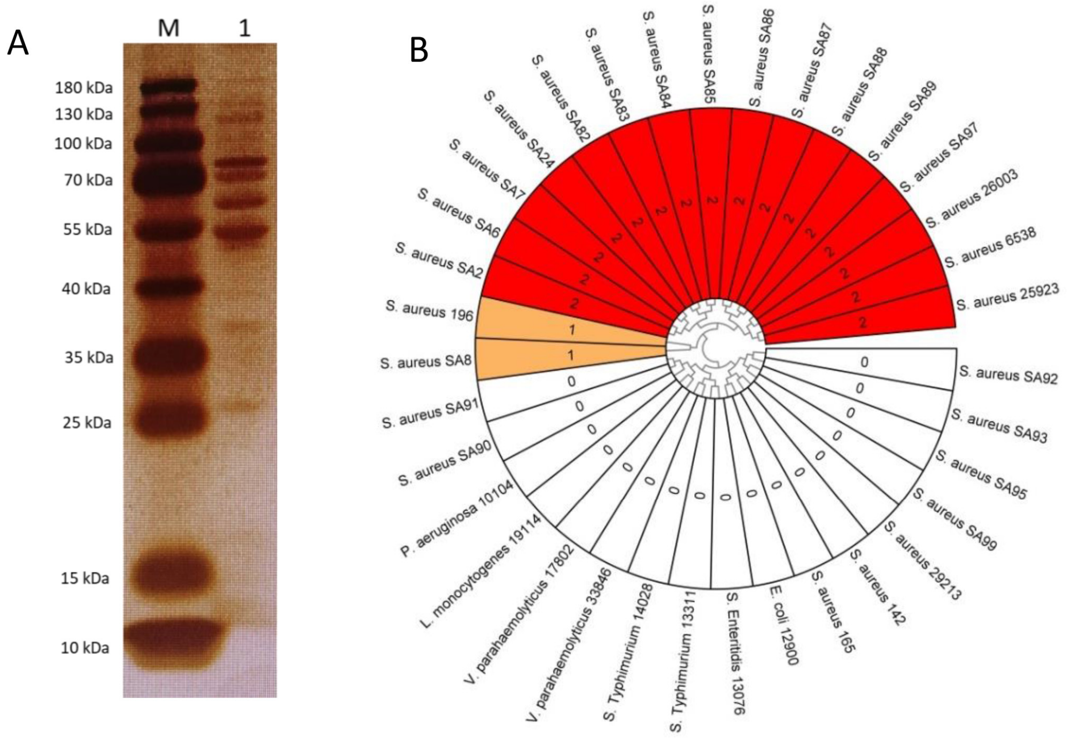

Figure S1. (A) Structural protein analysis by SDS-PAGE. M: marker; lane 1: phage LSA2302. (B) The host range of phage LSA2302 with scoring on lytic zones: "2" denotes strong intensity (clear plaque); "1" denotes weak intensity (opaque plaque); "0" denotes no lytic activity (no plaque).
