# Supplementary material for: Phage-Based Biosensing for Rapid and Specific Detection of Staphylococcus aureus
Source: Microorganisms. 2023 Aug 17;11(8):2098. doi: 10.3390/microorganisms11082098 (PMC10458643; doi:10.3390/microorganisms11082098)
Supplement: Supplementary file 1 [file microorganisms-11-02098-s001.zip › Table S1.pdf]

Table S1 Bacterial strains used in this study and the host range of phage LSA2302.

Number	G+/G-	Strains	Source	Species	Lytic ability
1	G+	SA2	Lab Collection	*Staphylococcus aureus*	2
2	G+	SA6	Lab Collection	*Staphylococcus aureus*	2
3	G+	SA7	Lab Collection	*Staphylococcus aureus*	2
4	G+	SA8	Lab Collection	*Staphylococcus aureus*	1
5	G+	SA24	Lab Collection	*Staphylococcus aureus*	2
6	G+	SA82	Lab Collection	*Staphylococcus aureus*	2
7	G+	SA83	Lab Collection	*Staphylococcus aureus*	2
8	G+	SA84	Lab Collection	*Staphylococcus aureus*	2
9	G+	SA85	Lab Collection	*Staphylococcus aureus*	2
10	G+	SA86	Lab Collection	*Staphylococcus aureus*	2
11	G+	SA87	Lab Collection	*Staphylococcus aureus*	2
12	G+	SA88	Lab Collection	*Staphylococcus aureus*	2
13	G+	SA89	Lab Collection	*Staphylococcus aureus*	2
14	G+	SA90	Lab Collection	*Staphylococcus aureus*	0
15	G+	SA91	Lab Collection	*Staphylococcus aureus*	0
16	G+	SA92	Lab Collection	*Staphylococcus aureus*	0
17	G+	SA93	Lab Collection	*Staphylococcus aureus*	0
18	G+	SA95	Lab Collection	*Staphylococcus aureus*	0
19	G+	SA97	Lab Collection	*Staphylococcus aureus*	2
20	G+	SA99	Lab Collection	*Staphylococcus aureus*	0
21	G+	29213	ATCC [a]	*Staphylococcus aureus*	0
22	G+	26003	CMCC [b]	*Staphylococcus aureus*	2
23	G+	6538	ATCC	*Staphylococcus aureus*	2
24	G+	25923	ATCC	*Staphylococcus aureus*	2
25	G+	196	Lab Collection	*Staphylococcus aureus*	1
26	G+	142	Lab Collection	*Staphylococcus aureus*	0
27	G+	165	Lab Collection	*Staphylococcus aureus*	0
28	G-	12900	ATCC	*Escherichia coli* O157:H7	0
29	G-	13076	ATCC	*Salmonella* Enteritidis	0
30	G-	13311	ATCC	*Salmonella* Typhimurium	0
31	G-	14028	ATCC	*Salmonella* Typhimurium	0
32	G-	33846	ATCC	*Vibrio parahaemolyticus*	0
33	G-	17802	ATCC	*Vibrio parahaemolyticus*	0
34	G-	19114	ATCC	*Listeria monocytogenes*	0
35	G-	10104	CMCC	*Pseudomonas aeruginosa*	0

2, strong intensity (Clear plaque); 1, weak intensity (Opaque plaque); 0, no lytic activity (No plaque).

ATCC [a], American Type Culture Collection. CMCC [b], Center for Medical Culture Collections.
