# Supplementary material for: Phage-Based Biosensing for Rapid and Specific Detection of Staphylococcus aureus
Source: Microorganisms. 2023 Aug 17;11(8):2098. doi: 10.3390/microorganisms11082098 (PMC10458643; doi:10.3390/microorganisms11082098)
Supplement: Supplementary file 1 [file microorganisms-11-02098-s001.zip › Table S2.pdf]

Table S2 The optimal multiplicity of infection (MOI) of phage LSA2302.

Phage concentration (PFU/mL)	Bacterial concentration (CFU/mL)	Multiplicity of infection (MOI)	Titer (PFU/mL)
10^4	10^6	0.01	1.3×10^8
10^5	10^6	0.1	1.4×10^8
10^6	10^6	1	1.6×10^8
10^7	10^6	10	3.05×10^8
10^8	10^6	100	2.75×10^8
