# Supplementary material for: Phage-Based Biosensing for Rapid and Specific Detection of Staphylococcus aureus
Source: Microorganisms. 2023 Aug 17;11(8):2098. doi: 10.3390/microorganisms11082098 (PMC10458643; doi:10.3390/microorganisms11082098)
Supplement: Supplementary file 1 [file microorganisms-11-02098-s001.zip › Table S3.pdf]

Table S3 Genomic features of phage LSA2302.

Features	LSA2302
Genomic size (bp)	141325
No. ORFs	222
No. Hypothetical gene	133
G+C content (%)	30.20%
No. tRNA	3
No. Promoter	23
No. Terminators	70
