# Supplementary material for: Phage-Based Biosensing for Rapid and Specific Detection of Staphylococcus aureus
Source: Microorganisms. 2023 Aug 17;11(8):2098. doi: 10.3390/microorganisms11082098 (PMC10458643; doi:10.3390/microorganisms11082098)
Supplement: Supplementary file 1 [file microorganisms-11-02098-s001.zip › Table S4.pdf]

Table S4 Predicted ORFs in the phage LSA2302 genome.

ORF	Start	Stop	Strand	Predicted molecular function
1	293	72	-	hypothetical protein
2	1165	785	-	hypothetical protein
3	1588	1265	-	TreP
4	1912	1754	-	TreN
5	2637	2446	-	terminal repeat-encoded protein
6	2974	2687	-	TreK
7	3488	3180	-	TreJ
8	3942	4226	+	hypothetical protein
9	5357	5121	-	hypothetical protein
10	5693	5445	-	hypothetical protein
11	5876	5694	-	TreE
12	6280	5987	-	terminal repeat-encoded protein
13	6699	6391	-	hypothetical protein
14	6990	6700	-	TreC
15	7284	7111	-	membrane protein
16	7574	7281	-	TreA
17	8437	7775	-	hypothetical protein
18	8797	8483	-	hypothetical protein
19	9423	9322	-	hypothetical protein
20	9627	9460	-	hypothetical protein
21	9964	9689	-	hypothetical protein
22	10220	10002	-	hypothetical protein
23	10555	10220	-	hypothetical protein
24	10713	10552	-	hypothetical protein
25	10942	10703	-	hypothetical protein
26	11241	10942	-	hypothetical protein
27	11558	11247	-	hypothetical protein
28	11821	11624	-	hypothetical protein
29	12215	11823	-	hypothetical protein
30	12480	12235	-	hypothetical protein
31	14028	12559	-	putative nicotinamide phosphoribosyl transferase
32	14954	14046	-	putative ribose-phosphate pyrophosphokinase
33	15244	14951	-	hypothetical protein
34	15627	15337	-	hypothetical protein
35	15828	15628	-	hypothetical protein
36	16065	15841	-	hypothetical protein
37	16239	16081	-	putative membrane protei
38	16458	16255	-	hypothetical protein
39	17189	16536	-	hypothetical protein
40	17507	17190	-	hypothetical protein
41	17773	17510	-	hypothetical protein

42	17967	17788	-	hypothetical protein
43	18324	18013	-	hypothetical protein
44	18832	18305	-	metallophosphatase
45	19065	18829	-	hypothetical protein
46	19483	19076	-	hypothetical protein
47	19788	19483	-	hypothetical protein
48	20133	19864	-	hypothetical protein
49	20239	20126	-	hypothetical protein
50	20537	20250	-	hypothetical protein
51	20847	20554	-	hypothetical protein
52	21259	20849	-	putative membrane protein
53	21664	21485	-	hypothetical protein
54	22280	21678	-	hypothetical protein
55	22630	22280	-	hypothetical protein
56	22950	22645	-	hypothetical protein
57	23298	LSA23020	-	putative membrane protein
58	23645	23298	-	hypothetical protein
59	24026	23658	-	hypothetical protein
60	24221	24039	-	putative membrane protein
61	24565	24269	-	hypothetical protein
62	24734	24558	-	hypothetical protein
63	24994	24746	-	hypothetical protein
64	25654	25010	-	ribulose-1,5-bisphosphate carboxylase/oxygenase small subunit
65	25968	25735	-	hypothetical protein
66	26212	25961	-	hypothetical protein
67	26378	26202	-	hypothetical protein
68	26971	26414	-	hypothetical protein
69	27218	26976	-	hypothetical protein
70	27763	27365	-	putative membrane protein
71	28529	27825	-	hypothetical protein
72	28989	28546	-	hypothetical protein
73	29512	29054	-	hypothetical protein
74	30261	29530	-	hypothetical protein
75	31496	30633	-	hypothetical protein
76	31942	31496	-	hypothetical protein
77	32687	31920	-	hypothetical protein
78	33216	32680	-	hypothetical protein
79	33591	33280	-	hypothetical protein
80	33946	33578	-	membrane protein
81	35210	33960	-	putative DNA repair exonuclease
82	35958	35203	-	hypothetical protein
83	36222	35962	-	major tail protein
84	36544	36317	-	putative major tail protein

85	37071	36559	-	tail protein
86	37725	37093	-	putative Ig-like protein
87	38515	37853	-	RNA polymerase sigma facto
88	38855	38502	-	hypothetical protein
89	40115	38859	-	putative DNA repair protein
90	41446	40175	-	hypothetical protein
91	42015	41533	-	hypothetical protein
92	42274	42032		hypothetical protein
93	45562	42344	-	DNA polymerase I
94	45943	45638	-	HU family DNA-binding protein
95	46549	45953		hypothetical protein
96	47077	46757	-	thioredoxin-like protein
97	47390	47061	-	hypothetical protein
98	48457	47408	-	ribonucleoside-diphosphate beta subunit
99	50585	48471	-	putative ribonucleotide reductase large subunit
100	50992	50600	-	ribonucleotide reduction protein NrdI
101	51618	51010	-	resolvase
102	52057	51605	-	hypothetical protein
103	52395	52057	-	hypothetical protein
104	53527	52460	-	DNA primase
105	54138	53542	-	hypothetical protein
106	56057	54138	-	recombination endonuclease
107	56434	56057	-	hypothetical protein
108	57459	56434	-	recombination exonuclease
109	57878	57459	-	acetyltransferase
110	59399	57957	-	helicase DnaB-like
111	61005	59392	-	replication protein
112	62765	61017	-	DNA helicase
113	64231	62855	-	putative tail fiber protein
114	64612	64238	-	hypothetical protein
115	66548	64626	-	capsid and scaffold protein
116	66707	64549	-	hypothetical protein
117	70214	66756	-	adsorption-associated tail protein
118	70756	70235	-	baseplate wedge protein
119	73932	70867		hypothetical protein
120	74999	73953	-	baseplate J-like protein
121	75718	75014	-	putative baseplate protein
122	76242	75718	-	hypothetical protein
123	77033	76242	-	hypothetical protein
124	79686	77140	-	putative glycerophosphoryl diester phosphodiesterase
125	80573	79686	-	peptidoglycan hydrolase
126	83013	80587	-	N-acetylmuramoyl-L-alanine amidase
127	87149	83091	-	tail tape measure

128	87738	87202	-	RNA polymerase
129	88240	87782	-	tail tape measure protein
130	88683	88372	-	hypothetical protein
131	88948	88754	-	hypothetical protein
132	89413	88961	-	hypothetical protein
133	89596	89456	-	hypothetical protein
134	89744	89586	-	hypothetical protein
135	90262	89834	-	tail tube protein
136	92098	90335	-	tail sheath protein
137	92340	92125	-	hypothetical protein
138	93178	92342	-	hypothetical protein
139	93817	93197	-	hypothetical protein
140	94695	93817	-	capsid protein
141	95617	94709	-	hypothetical protein
142	95926	95630	-	hypothetical protein
143	97409	96018	-	major capsid protein
144	98484	97525	-	hypothetical protein
145	99276	98503	-	prohead protease
146	101162	99471	-	portal protein
147	101537	101166	-	putative portal protein
148	103151	101982	-	putative membrane protein
149	103723	103244	-	membrane protein
150	103893	103720	-	hypothetical protein
151	104701	103880	-	structural head protein
152	106511	104694	-	terminase large subunit
153	106936	106526	-	hypothetical protein
154	107211	106933	-	hypothetical protein
155	107455	107189	-	membrane protein
156	107716	107982	-	hypothetical protein
157	108015	108341	+	membrane protein
158	108354	108686	+	hypothetical protein
159	108699	108908	+	hypothetical protein
160	109389	109607	+	hypothetical protein
161	111150	111335	+	hypothetical protein
162	111420	111923	+	putative holin
163	111923	113410	+	lysin
164	113524	113832	+	membrane protein
165	113832	114626	+	membrane protein
166	114823	115515	+	putative transglycosylase
167	115624	115851	+	hypothetical protein
168	115854	116084	+	transcriptional regulator
169	116074	116715	+	hypothetical protein
170	116738	116929	+	hypothetical protein
171	116919	117344	+	putative ribonuclease

172	117360	117974	+	hypothetical protein
173	118026	118766	+	putative PhoH-related protein
174	118834	119058	+	hypothetical protein
175	119062	119955	+	RNA ligase
176	119948	120574	+	nucleoside 2-deoxyribosyltransferase
177	120567	121145	+	hypothetical protein
178	121152	121325	+	LysM motif protein
179	121342	121605	+	hypothetical protein
180	121685	123736	+	hypothetical protein
181	123737	123898	+	hypothetical protein
182	123942	124130	+	hypothetical protein
183	124130	124432	+	NTP pyrophosphohydrolase
184	124565	124981	+	putative nucleotide kinase
185	124974	125300	+	hypothetical protein
186	125453	126571	+	putative porphyrin biosynthetic protein
187	126584	127429	+	AAA family ATPase
188	127429	127707	+	putative membrane protein
189	127710	127874	+	hypothetical protein
190	127877	128407	+	hypothetical protein
191	128407	128949	+	hypothetical protein
192	128999	129481	+	hypothetical protein
193	129522	129695	+	putative transglycosylase
194	129789	130178	+	hypothetical protein
195	130180	130419	+	hypothetical protein
196	130431	130535	+	hypothetical protein
197	130598	131335	+	hypothetical protein
198	131325	131519	+	hypothetical protein
199	131520	131738	+	hypothetical protein
200	131742	132290	+	hypothetical protein
201	133079	133780	+	serine/threonine protein phosphatase
202	133783	134187	+	hypothetical protein
203	134200	134694	+	GTP cyclohydrolase II
204	134710	135141	+	hypothetical protein
205	135147	135605	+	hypothetical protein
206	135605	135859	+	hypothetical protein
207	135852	136337	+	hypothetical protein
208	136334	136525	+	hypothetical protein
209	136525	136779	+	hypothetical protein
210	136772	137326	+	hypothetical protein
211	137326	137736	+	hypothetical protein
212	137749	137994	+	hypothetical protein
213	138008	138256	+	BofL
214	138346	138636	+	hypothetical protein
215	139125	138946	-	hypothetical protein

216	139479	139210	-	terminal repeat-encoded protein
217	139652	139479	-	terminal repeat-encoded protein
218	139919	139656	-	hypothetical protein
219	140460	139990	-	terminal repeat-encoded protein
220	140776	140540	-	hypothetical protein
221	141031	140867	-	hypothetical protein
222	141239	141102	-	hypothetical protein
