# Supplementary material for: Phage-Based Biosensing for Rapid and Specific Detection of Staphylococcus aureus
Source: Microorganisms. 2023 Aug 17;11(8):2098. doi: 10.3390/microorganisms11082098 (PMC10458643; doi:10.3390/microorganisms11082098)
Supplement: Supplementary file 1 [file microorganisms-11-02098-s001.zip › Table S5.pdf]

Table S5 The comparison of pMB-based biosensing with other previously reported methods for *Staphylococcus aureus* detection.

Method	Linear range (CFU/mL)	LOD	Time	Ref.
Colorimetric detection	10^1-10^4	0.2 CFU/mL	100 min	[1]
Fluorescence detection	7.22×10^0-1.44×10^9	4 CFU/mL	in 120 min	[2]
Dark-field light scattering imaging with plasma biosensors	\	8×10^4 CFU/mL	15-20 min	[3]
Phagomagnetic separation with immunoassay	1.0×10^4-1.0×10^6	10^3 CFU/mL	in 90 min	[4]
Strand exchange amplification	\	10^0 CFU/g	60-120 min	[5]
Magnetic separation with mPCR	\	100 CFU/mL	240 min	[6]
Magnetic separation with bioluminescence	2.3×10^3-1.2×10^7	2.2×10^2 CFU/mL	in 33 min	[7]
Magnetic separation with bioluminescence	1.0×10^2-1.0×10^7	33 CFU/mL	in 70 min	[8]
Immunomagnetic with enzyme linked	\	4×10^3 CFU/mL	90 min	[9]
Magnetic with fluorescence detection	1.0×10^2-1.0×10^7	70 CFU/mL	less than 50 min	[10]
Fluorescence-enhanced lateral flow biosensor	\	5.4×10^2 CFU/mL	in 70 min	[11]
Nanoflower-based ELISA method	10^1-10^6	6 CFU/mL	over 240 min	[12]
Electrochemical aptasensor	10-10^8	3 CFU/mL	15 min	[13]
Electrochemical aptasensor	1.2×10^1-1.2×10^8	1 CFU/mL	30 min	[14]
Immunomagnetic with colorimetric detection	10-10^6	2.4 CFU/mL	about 35 min	[15]
Long-period fiber grating immunosensor	10^2-10^7	33 CFU/mL	20 min	[16]
Biolayer interferometry technology	\	13 CFU/mL	16 min	[17]
Magnetic separation with colorimetric	10^1-10^6	3.0×10^2 CFU/mL	120 min	[18]
Magnetic separation with surface-enhanced Raman scattering	7.6×10^1-7.6×10^7	1.09 CFU/mL	50 min	[19]
Magnetic with fluorescence detection	10^2-10^5	2.7×10^2 CFU/mL	80 min	[20]
Magnetic with fluorescence detection	3×10^1-3×10^6	30 CFU/mL	45 min	[21]
Molecular imprinted aptasensor	10^1-10^7	1 CFU/mL	more than 110 min	[22]

Sandwich lateral flow assay	1.0×10^3-1.0×10^8	1.0×10^3 CFU/mL	about 40 min	[23]
Phage amplification with multiplex qPCR	10-10^8	10 CFU/mL	no more than 240 min	[24]
Sandwich ELISA	\	1.4×10^5 CFU/mL	more than 180 min	[25]
CRISPR-Cas13a based bacterial detection	10^0-10^7	1 CFU/mL	4 h	[26]
High throughput colorimetric biosensor	10^2-10^7	81 CFU/mL	5.5 h	[27]
Polymerase spiral reaction with visual detection	\	1.99×10^3 CFU/g	2 h	[28]
This work	1×10^4-1×10^8	2.43×10^3 CFU/mL	in 30 min	\

References:

1. Sun, R., et al., *Vancomycin recognition and induced-aggregation of the Au nanoparticles through freeze-thaw for foodborne pathogen Staphylococcus aureus detection.* 2022(1873-4324 (Electronic)).
2. Du, H.A.-O., et al., *Sandwich Fluorescence Detection of Foodborne Pathogen Staphylococcus aureus with CD Fluorescence Signal Amplification in Food Samples. LID - 10.3390/foods11070945 [doi] LID - 945.* 2022(2304-8158 (Print)).
3. Imai M, M.K., Tomonari H , Jumpei Uchiyama, J., et al., *Dark-Field Microscopic Detection of Bacteria using Bacteriophage-Immobilized SiO(2)@AuNP Core-Shell Nanoparticles.* 2019(1520-6882 (Electronic)).
4. Yan, C., et al., *Combining phagomagnetic separation with immunoassay for specific, fast and sensitive detection of Staphylococcus aureus.* 2017(1873-3573 (Electronic)).
5. Liu, C., et al., *Rapid and Simple Detection of Viable Foodborne Pathogen Staphylococcus aureus.* 2019(2296-2646 (Print)).
6. Bai, X., et al., *Simultaneous detection of Bacillus cereus and Staphylococcus aureus by teicoplanin functionalized magnetic beads combined with triplex PCR.* Food Control, 2022. **132**: p. 108531.
7. Fan, E., et al., *Quantification of live Gram-positive bacteria via employing artificial antibacterial peptide-coated magnetic spheres as isolation carriers.* Microchemical Journal, 2020. **154**: p. 104643.
8. Yu, J., et al., *Sensitive and rapid detection of staphylococcus aureus in milk via cell binding domain of lysin.* 2016(1873-4235 (Electronic)).
9. Yi, Z., et al., *Lysin cell-binding domain-functionalized magnetic beads for detection of Staphylococcus aureus via inhibition of fluorescence of Amplex Red/hydrogen peroxide assay by intracellular catalase.* 2019(1618-2650 (Electronic)).
10. Yi, Z., et al., *Lysin cell-binding domain-functionalized magnetic beads for detection of Staphylococcus aureus via inhibition of fluorescence of Amplex Red/hydrogen*

peroxide assay by intracellular catalase. Analytical and Bioanalytical Chemistry, 2019. **411**(27): p. 7177-7185.

11. Zhou, B., et al., *CRISPR/Cas12a based fluorescence-enhanced lateral flow biosensor for detection of Staphylococcus aureus.* Sensors and Actuators B: Chemical, 2022. **351**: p. 130906.
12. Yin, W., et al., *Bio-hybrid nanoarchitectonics of nanoflower-based ELISA method for the detection of Staphylococcus aureus.* Sensors and Actuators B: Chemical, 2022. **366**: p. 132005.
13. Sohouli, E., et al., *A new electrochemical aptasensor based on gold/nitrogen-doped carbon nano-onions for the detection of Staphylococcus aureus.* Electrochimica Acta, 2022. **403**: p. 139633.
14. Ranjbar, S. and S. Shahrokhian, *Design and fabrication of an electrochemical aptasensor using Au nanoparticles/carbon nanoparticles/cellulose nanofibers nanocomposite for rapid and sensitive detection of Staphylococcus aureus.* Bioelectrochemistry, 2018. **123**: p. 70-76.
15. Liu, Y., et al., *A colorimetric sensor for Staphylococcus aureus detection based on controlled click chemical-induced aggregation of gold nanoparticles and immunomagnetic separation.* Microchimica Acta, 2022. **189**(3): p. 104.
16. Gan, W., et al., *Rapid and sensitive detection of Staphylococcus aureus by using a long-period fiber grating immunosensor coated with egg yolk antibody.* Biosensors and Bioelectronics, 2022. **199**: p. 113860.
17. Liu, X., et al., *Rapid and sensitive detection of Staphylococcus aureus using biolayer interferometry technology combined with phage lysin LysGH15.* Biosensors and Bioelectronics, 2022. **198**: p. 113799.
18. Wang, Z., et al., *An integrated system using phenylboronic acid functionalized magnetic beads and colorimetric detection for Staphylococcus aureus.* Food Control, 2022. **133**: p. 108633.
19. Qi, X., et al., *An ultrasensitive and dual-recognition SERS biosensor based on Fe3O4@Au-Teicoplanin and aptamer functionalized Au@Ag nanoparticles for detection of Staphylococcus aureus.* Talanta, 2022. **250**: p. 123648.
20. Wang, Z., et al., *A novel PEG-mediated boric acid functionalized magnetic nanomaterials based fluorescence biosensor for the detection of Staphylococcus aureus.* Microchemical Journal, 2022. **178**: p. 107379.
21. Huang, J., et al., *A Dual-Recognition Strategy for Staphylococcus aureus Detection Using Teicoplanin-Modified Magnetic Nanoparticles and IgG-Functionalized Quantum Dots.* Food Analytical Methods, 2022. **15**(7): p. 1968-1978.
22. El-Wekil, M.M., et al., *An innovative dual recognition aptasensor for specific detection of Staphylococcus aureus based on Au/Fe3O4 binary hybrid.* Scientific Reports, 2022. **12**(1): p. 12502.
23. Zhao, M., et al., *Antibiotic and mammal IgG based lateral flow assay for simple and sensitive detection of Staphylococcus aureus.* Food Chemistry, 2021. **339**: p.

127955.
24. Huang, C., et al., *Dual phage amplification-mediated multiplex detection strategies for the simultaneous detection of Salmonella enterica and Staphylococcus aureus.* Talanta, 2023. **253**: p. 124095.
25. Hu, Y., et al., *Selection of specific nanobodies to develop an immuno-assay detecting Staphylococcus aureus in milk.* Food Chemistry, 2021. **353**: p. 129481.
26. Zhou, J., et al., *CRISPR-Cas13a based bacterial detection platform: Sensing pathogen Staphylococcus aureus in food samples.* Analytica Chimica Acta, 2020. **1127**: p. 225-233.
27. Yu, T., et al., *Aptamer based high throughput colorimetric biosensor for detection of staphylococcus aureus.* Scientific Reports, 2020. **10**(1): p. 9190.
28. Milton, A.A.P., et al., *Development of a novel polymerase spiral reaction (PSR) assay for rapid and visual detection of Staphylococcus aureus in meat.* LWT, 2021. **139**: p. 110507.
